# Abnormal Resting-State Quantitative Electroencephalogram in Children With Central Auditory Processing Disorder: A Pilot Study

**DOI:** 10.3389/fnins.2018.00292

**Published:** 2018-05-11

**Authors:** Rafał Milner, Monika Lewandowska, Małgorzata Ganc, Elżbieta Włodarczyk, Diana Grudzień, Henryk Skarżyński

**Affiliations:** ^1^Department of Experimental Audiology, World Hearing Center, Institute of Physiology and Pathology of Hearing, Warsaw, Poland; ^2^Bioimaging Research Center, World Hearing Center, Institute of Physiology and Pathology of Hearing, Warsaw, Poland; ^3^Faculty of Humanities, Nicolaus Copernicus University, Toruń, Poland; ^4^Audiology and Phoniatrics Clinic, World Hearing Center, Institute of Physiology and Pathology of Hearing, Warsaw, Poland; ^5^Rehabilitation Clinic, World Hearing Center, Institute of Physiology and Pathology of Hearing, Warsaw, Poland; ^6^World Hearing Center, Institute of Physiology and Pathology of Hearing, Warsaw, Poland

**Keywords:** central auditory processing disorder (CAPD), resting-state bioelectrical activity, quantitative electroencephalography (QEEG), central auditory processes, auditory deficits, children

## Abstract

In this study, we showed an abnormal resting-state quantitative electroencephalogram (QEEG) pattern in children with central auditory processing disorder (CAPD). Twenty-seven children (16 male, 11 female; mean age = 10.7 years) with CAPD and no symptoms of other developmental disorders, as well as 23 age- and sex-matched, typically developing children (TDC, 11 male, 13 female; mean age = 11.8 years) underwent examination of central auditory processes (CAPs) and QEEG evaluation consisting of two randomly presented blocks of “Eyes Open” (EO) or “Eyes Closed” (EC) recordings. Significant correlations between individual frequency band powers and CAP tests performance were found. The QEEG studies revealed that in CAPD relative to TDC there was no effect of decreased delta absolute power (1.5–4 Hz) in EO compared to the EC condition. Furthermore, children with CAPD showed increased theta power (4–8 Hz) in the frontal area, a tendency toward elevated theta power in EO block, and reduced low-frequency beta power (12–15 Hz) in the bilateral occipital and the left temporo-occipital regions for both EO and EC conditions. Decreased middle-frequency beta power (15–18 Hz) in children with CAPD was observed only in the EC block. The findings of the present study suggest that QEEG could be an adequate tool to discriminate children with CAPD from normally developing children. Correlation analysis shows relationship between the individual EEG resting frequency bands and the CAPs. Increased power of slow waves and decreased power of fast rhythms could indicate abnormal functioning (hypoarousal of the cortex and/or an immaturity) of brain areas not specialized in auditory information processing.

## Introduction

Central auditory processing disorder (CAPD) refers to a dysfunction in how the central nervous system (CNS) utilizes auditory information. CAPD is recognized when the peripheral hearing is normal, but there are deficits in one or more of the following central auditory processes (CAPs): sound source localization; auditory stimuli discrimination; recognition of acoustic patterns (temporal patterning); temporal aspects of audition, including temporal integration, temporal discrimination (e.g., gap detection), temporal ordering/sequencing of rapid events and temporal masking; auditory performance in competing acoustic signals (including dichotic listening) or understanding of degraded speech (ASHA, 2005; American Academy of Audiology, 2010; British Society of Audiology, 2011a). These abnormal CAPs are believed to result from CNS lesions in the areas specialized in processing of auditory stimuli and responsible for interhemispheric transfer of acoustic information (Jerger et al., 2002; Moncrieff, 2006) as well as from several factors such as premature birth, low birth weight and chronic ear infections. The prevalence of CAPD in school-aged children has not been firmly established. Chermak and Musiek (1997) estimated the prevalence of CAPD to be 2–3% with a 2:1 ratio between boys and girls, whereas Santucci (2003) reported the prevalence of CAPD in the pediatric population to be ~3–5%.

Various symptoms and an unrecognized brain dysfunctions associated with CAPD entail a lack of consensus regarding the definition of CAPD (Dillon, 2012; Moore and Hunter, 2013; Moore et al., 2013). It has been recommended to include in the CAPD assessment a clinical history of the patient (Micallef, 2016), well-validated questionnaires to recognize real-life listening difficulties (Moore et al., 2013), and standardized verbal and non-verbal tests for measuring central auditory processing (Micallef, 2016). However, presently there is neither universally accepted CAP test battery nor specific cut-offs for CAPD evaluation.

CAPD assessment becomes even more problematic when we consider that listening difficulties often coexist with language and/or attention disorders including the attention deficit disorder (ADD) and/or the attention-deficit/hyperactivity disorder (ADHD) (Keith and Engineer, 1991; Riccio et al., 1994, 1996), dyslexia (Cacace and McFarland, 2006; Zaidan and Baran, 2013), and specific language impairment (SLI) (Sharma et al., 2009; Włodarczyk et al., 2015). For example, DiMaggio and Geffiner (2003) demonstrated that 84% of children with CAPD fulfilled the diagnostic criteria for the ADHD. In this study the concomitance of ADHD and CAPD was 41% for children with confirmed diagnosis of ADHD and 43% for children suspected of having ADHD.

The comorbidity of ADHD and CAPD symptoms has encouraged researchers to investigate whether there are any auditory deficits that would delineate ADHD subtypes. The research in this area indicates that children with the ADD with and without hyperactivity exhibit different auditory symptoms (Chermak et al., 2002; Øie et al., 2014; Serrallach et al., 2016). The ADD with hyperactivity is thought to coexist with poor performance in tasks requiring temporal ordering (Fostick, 2017) or temporal patterning, dichotic listening and understanding of speech in the presence of background noise (Lanzetta-Valdo et al., 2017). In another study, children with ADD with hyperactivity showed poor performance in melodic and rhythm processing tasks, whereas their peers diagnosed with ADD had no such impairments (Serrallach et al., 2016). In children diagnosed with the predominantly inattentive ADHD subtype, the auditory divided attention deficits and difficulty in listening speech in the presence of background noise have been reported by Chermak and Musiek (1992). Interestingly, in a more recent study the predominantly hyperactive and combined (inattentive and hyperactive) ADHD subtypes showed different abnormal patterns in a dichotic speech listening task (Øie et al., 2014). Some authors, however (e.g., Ghanizadeh, 2009) failed to demonstrate that ADHD subgroups could be differentiated based on the auditory processing deficits.

A lack of consensus about the diagnostic criteria for CAPD has motivated researchers and clinicians to seek objective methods for the evaluation processes. Audiological societies (American Academy of Audiology, 2010; British Society of Audiology, 2011a) recommend the use of auditory evoked potentials (AEPs) such as the auditory brainstem response (ABR), middle latency responses (MLR), and cortical auditory evoked responses (CAEP) to evaluate CAPD more objectively. ABR, MLR, and CAEP test information processing at subsequent levels of the auditory pathway, from the auditory nerve to the primary auditory cortex and higher cortical areas. It has been shown that children with CAPD have atypical ABR and/or MLR (Schochat et al., 2014; for a review Abdollahi et al., 2017), which may indicate impaired information processing at the lower levels of the central auditory system. Previous CAEP studies have demonstrated the abnormal functioning of the primary and/or secondary auditory cortex in CAPD (Sharma et al., 2014; Tomlin and Rance, 2016; Koravand et al., 2017). Specifically, children with listening difficulties had longer latencies and smaller amplitudes of the early components of CAEP, P1, and N1 (Sharma et al., 2014; Tomlin and Rance, 2016), as well as prolonged latencies of N2 (Koravand et al., 2017) compared to their normally developing peers. All these CAEPs are thought to be elicited in the auditory cortices (Bruneau and Gomot, 1998; Ponton et al., 2002). Later CAEPs, such as the Mismatch Negativity (MMN), considered to be a pre-attentive response to a change in repetitive acoustic stimulation (Näätänen et al., 1978), and the P300, reflecting attention and/or working memory engagement in auditory information processing (Polich, 2007), have also been extensively investigated in children and adults with CAPD (Liasis et al., 2003; Sharma et al., 2006; Roggia and Colares, 2008; Koravand et al., 2017). In contrast to MMN, which is generated in the auditory cortex (Giard et al., 1990; Alho, 1995), P300 source is located in the non-auditory areas (Baudena et al., 1995; Halgren et al., 1995; Friedman, 2003; Van Dinteren et al., 2018). The evidence of abnormal MMN in children with CAPD is inconsistent: one study reported lower incidence and longer latency of MMN in the CAPD group compared to normally developing controls (Bauer et al., 2009) while others found no significant abnormalities (Liasis et al., 2003; Roggia and Colares, 2008; Koravand et al., 2017). On the other hand, P300 latencies were found to be longer in children with CAPD than in their counterparts without listening problems (Jirsa and Clontz, 1990). Furthermore, both P300 latency and amplitude have been shown to be useful tools to monitor the effectiveness of CAPD treatment (Jirsa, 1992; Alonso and Schochat, 2009).

Unlike the ABR, the CAEP are strongly influenced by the subject's state (e.g., arousal level, motivation, fatigue). In addition, abnormal changes in the parameters of some CAEP tests are not specific for a particular dysfunction, e.g., reduced amplitude and prolonged latency of P300 (one of the CAEP components) could be well-observed in dementia (Polich et al., 1986), alcoholism (Lewis et al., 2013), and different neurological (Hansch et al., 1982) or psychiatric disorders (Blum et al., 1994). Hence, there is still a considerable need to establish an objective method to evaluate CAPD.

Advanced neuroimaging techniques such as functional magnetic resonance imaging (fMRI) (Schmithorst et al., 2011; Farah et al., 2014; Pluta et al., 2014), CAEP-fMRI (Rusiniak et al., 2013; Milner et al., 2014), MRI/MEG (Serrallach et al., 2016), and positron emission tomography (PET) (Kim et al., 2009) provide insights into the brain representation of CAPs. Application of these techniques could significantly improve the current diagnostics of listening difficulties. Activation patterns in normal hearing individuals during CAP tests that are modified and adapted for fMRI may provide a good reference for the CAPD assessment (Bartel-Friedrich et al., 2010). Neuroimaging studies using dichotic listening paradigms have revealed the dominance of left hemispheric areas (the upper posterior plane of the temporal lobes, including the Heschl's gyrus and planum temporale) in perception of speech sounds in the normal human brain (Hugdahl and Westerhausen, 2016 for a review). Neural correlates of temporal processing of acoustic stimuli mainly involved the primary and secondary auditory cortices (Mitsudo et al., 2014). Neural representation of the temporal aspects of audition is, however, strongly dependent on the time scale being tested (Lewis and Miall, 2003; Szelag et al., 2009 for a review). Specifically, with respect to the subsecond durations, activations of the primary and secondary auditory cortices as well as the cerebellum and basal ganglia were postulated (Lewis and Miall, 2003). One of the typical tasks used in these studies is to detect a brief silent gap embedded in an ongoing auditory signal (Phillips et al., 2010). Subsecond timing may also be assessed by temporal order judgments, i.e., reporting the order of two consecutive sounds presented in rapid succession (Szymaszek et al., 2006, 2009; Szelag et al., 2014). This task predominantly activates the temporo-parietal junction (TPJ) (e.g., Bernasconi et al., 2010), which is located at the intersection of the posterior part of superior temporal sulcus, the inferior parietal lobule, and the lateral occipital cortex. In the suprasecond timescale, the recruitment of not only auditory cortices but also prefrontal and parietal areas as well as the corpus callosum was observed (e.g., Szelag et al., 2009). The involvement of the non-auditory brain areas in temporal processing of acoustic stimuli is thought to reflect increased cognitive (e.g., attentional or short-term memory) demands of these tasks.

Previous studies have shown that CAPD co-occurs with anatomical and/or functional brain abnormalities not only in the areas specialized in processing of acoustic information (Kim et al., 2009; Schmithorst et al., 2011; Owen et al., 2013; Farah et al., 2014; Pluta et al., 2014; Micallef, 2016 for a review). Kim et al. (2009) have found that the auditory cortex atrophy was accompanied by an increase in the metabolism of the bilateral Heschl's gyrus and precuneus in an adult patient with listening difficulties. Studies using the diffusion tensor imaging (DTI) technique showed decreased white matter integrity in the prefrontal cortex and left anterior cingulate in children with an abnormal left ear advantage in dichotic listening to speech stimuli (Farah et al., 2014). Furthermore, significant changes in the structures transmitting information between the thalamus and auditory cortex have been found in children with atypical hemispheric asymmetry in verbal sound processing (Farah et al., 2014).

A promising approach to investigation of the central auditory system is combining the objective methods of brain anatomical evaluation with psychoacoustic tests and measures of bioelectrical activity (Seither-Preisler et al., 2014; Serrallach et al., 2016). For example, children with ADHD, who often suffer from listening problems have smaller volume of bilateral Heschl's gyri and larger volumes of plana temporalia compared to their musically experienced peers (Seither-Preisler et al., 2014). Furthermore, the authors have also shown that in the ADHD the low values of Heschl's gyrus (primary auditory cortex)/planum temporale (secondary auditory cortex) ratio, calculated separately for both hemispheres, coexist with greater bilateral P1 component asynchrony (the P1 latency difference between the right and left auditory cortex) (Seither-Preisler et al., 2014). This brain-based marker may be used as a clinically relevant indicator of a higher risk of central auditory deficits in this group. The indices of interhemispheric asynchrony of the primary auditory-evoked P1 and N1 responses are also highly influenced by musical training (Kühnis et al., 2014; Serrallach et al., 2016). A combination of the primary-to-secondary auditory cortex ratio with the interhemispheric difference in the latency of P1 component and psychoacoustic test results can allow objective distinguishing between different developmental disorders with overlapping symptomatology such as ADHD, ADD, or dyslexia (Serrallach et al., 2016). As the auditory processing deficits often concur with other developmental disorders, the aforementioned neuronal marker may be a valuable tool in the assessment of listening problems.

In the present study, we examined the utility of quantitative analysis of electroencephalogram (QEEG) for diagnosing CAPD. QEEG converts the EEG data into frequency bands with the use of various mathematical algorithms [e.g., fast-Fourier transform (FFT), coherence analysis] and allows the calculation of the proportions of different frequency bands (Kropotov, 2009). QEEG gives the opportunity to define unique, highly valid, and reliable patterns of brain activity (Hughes and John, 1999; Thatcher, 2010). It also allows the calculation of coherence or co-modulation between specific EEG frequency bands recorded from different points on a patient's scalp, as well as between the sources of EEG estimated by different bioelectrical source localization algorithms [e.g., Low-Resolution Brain Electromagnetic Tomography (LORETA); Thatcher et al., 2007a,b; Thatcher, 2012]. QEEG is commonly used clinically to evaluate the outcomes of the neurofeedback training (a therapeutic method that teaches subjects to self-control brain functions by measuring brain waves and providing a feedback signal). Recently, the indices of intracerebral functional connectivity (IFC) and IFC-based neurofeedback have been proposed as a promising method of ameliorating the auditory-related dysfunctions (Elmer and Jäncke, 2014).

QEEG analysis has been most often applied to spontaneous EEG signals acquired at rest, when a patient is instructed to relax with eyes open or closed (Kaiser, 2007; Thatcher, 2011). Specific alterations in the resting-state EEG signals have been found in various neurodevelopmental disorders, especially ADHD (Arns et al., 2014; Roh et al., 2015; Chiarenza et al., 2016), ADD (Thompson and Thompson, 1998), dyslexia (Arns et al., 2007), and autism (Billeci et al., 2013). Evidence on resting-state brain activity in CAPD is scarce. Our preliminary fMRI study (Pluta et al., 2014) showed that children with listening difficulties had a reduced homogeneity in the default mode network (DMN) composed of the brain structures, showing an increased synchronization at wakeful rest and decreased correlation during a task (Raichle, 2015). We found inter-group differences in the precuneus/posterior cingulate and frontal pole, which are thought to be involved in the general attention processes (Ramnani and Owen, 2004; Cavanna, 2006). Therefore, based on these outcomes, we cannot conclude that there are specific areas responsible for attention related to listening.

The aim of the present study was two-fold: (1) to define the QEEG resting-state activity in children with CAPD and, if there would be a unique pattern owing to the core auditory deficits in this group, (2) to determine the relations between the individual frequency bands in this pattern and CAPs. Extraction of the QEEG activity specific to auditory dysfunctions will allow to consider this technique a supplementary method of evaluation of listening difficulties.

## Materials and methods

### Subjects

The CAPD study group consisted of 27 children (16 male, 11 female; mean age = 10.7 ± 2.1 years) with real-life listening problems reported by their parents and/or caregivers. These listening problems included: difficulty to follow utterances and performing verbal commands, distractibility, disorganization, poor concentration, and forgetting about daily activities. CAPD children were patients of the Institute of Physiology and Pathology of Hearing, recruited prior to their participation in a therapeutic program to improve auditory processing. The control group comprised 23 healthy age- and sex-matched typically developing children (TDC) (11 male, 13 female; mean age = 11.8 ± 2.3 years) whose parents and/or caregivers responded to advertisements in the local press.

All subjects had normal hearing, normal or corrected-to-normal vision, and no history of neuropsychiatric diseases or head trauma. They also did not take any medications affecting the CNS. All children attended school regularly and had intelligence within the normal range (Raven's progressive matrices). None of children had a formal diagnosis of dyslexia, SLI, autism, or ADHD and did not display any symptom of these disorders. However, it has been estimated that the accuracy of disease classification scales lies in the range between 47 and 79% (Tripp et al., 2006; Snyder et al., 2008). It practically means that we cannot with absolute certainty exclude the possibility that children with no official diagnosis of the above-listed developmental disorders were affected by attention and/or language deficits.

### Ethics approval

Caregivers/parents provided written informed consent for their children to participate in this study. The project was approved by the Ethics Committee of the Institute of Physiology and Pathology of Hearing and conformed to the tenets of the Declaration of Helsinki for medical research involving human subjects.

### Procedures

The study comprised audiological evaluation, administration of CAP, and attention batteries, and QEEG data acquisition. All procedures were performed at the Institute of Physiology and Pathology of Hearing in Warsaw, Poland. There were 2 sessions on 2 days within a week. On the first day, each subject participated in a medical interview and audiological examination followed by a CAP battery administration. On the second day, attention tests and an EEG study were performed. Each session lasted ~2 h (with short breaks). In the present study only the CAP battery and QEEG results were included.

### Audiological examination

The audiological examination included otoscopy, pure-tone audiometry (PTA), and impedance audiometry (IA), all conducted in accordance with standard procedures and compared to the normative values provided by the British Society of Audiology (British Society of Audiology, 2011b, 2013). The results of the PTA were treated as abnormal when hearing thresholds were > 20 dB HL on each frequency, in the range of 250–8,000 Hz in both ears. The IA results were considered abnormal when the middle ear pressure was < −150 mm of H_2_O pressure and compliance < 0.3 cc. The ABR tests allows the exclusion of study subjects with abnormal auditory functions of the brainstem pathways (e.g., children with auditory neuropathy) (Chermak and Musiek, 2007). The ABR procedure and outcomes calculation was performed in accordance with the American Clinical Neurophysiology Society Guideline (ACNS, 2006). The ABRs were recorded separately for each ear. An EEG active electrode was placed at the Fz position (10–20 standard; Jasper, 1958) and referenced/grounded to the mastoid of the same (A1) or the opposite (A2) ear, respectively. The impedance of recording electrodes was monitored and maintained below 5 kOhm. Clicks with alternating polarization presented at the 80-dB nHL intensity level and rate 31.1 stimuli/s via an ER3A insert earphone were used as stimuli to evoke ABRs. A total of 1,500 click repetitions were averaged for each ear. The correct morphology and amplitude ratio of waves I, III, and V were the criteria of the ABR evaluation. The ABRs with the highest amplitude of wave V, slightly lower amplitude of wave III, and the lowest amplitude of wave I were considered normal. Repeatability of the ABRs recorded with the same stimulation parameters, latency of individual waves, and the time intervals between individual peaks were also taken into account while evaluating the ABR accuracy. Responses with the interval between the waves I-III longer than between the waves III-V were considered to be correct. As the reference ranges for the individual ABR peak intervals were applied Polish norms proper for subjects' age developed by Kochanek (2000). Accordingly, the waves I with latency ≤ 1.9 ms and the waves V with latency ≤ 6.2 ms were considered as normal. Moreover, the I-III wave intervals ≤ 2.6 ms, III–V ≤ 2.4 ms, and I–V ≤ 4.6 ms were correct. An inter-ear difference between intervals (I–III and III–V) ≤ 0.2 ms, an inter-ear difference of waves V latency values ≤ 0.4 ms, and the amplitude ratio of the V and I waves ≥ 1.5 ms were taken as the correct values.

### CAP evaluation

In the present study, CAP cases were assessed by using the computerized battery developed by a joint research project between the Institute of Physiology and Pathology of Hearing and the Brigham Young University Department of Communication Disorders in the United States. The software was installed on a HP Compaq nx7400 laptop. Auditory stimuli were generated by a Creative SB1 100 external sound card (Creative Labs Inc., Jurong East, Singapore) and presented to the patient using Sennheiser HDA 200 headphones (Sennheiser, Wedemark, Germany). The following tests were administered: frequency pattern test (FPT), duration pattern test (DPT), gap detection test (GDT), dichotic digit test (DDT), and adaptive speech in noise (aSpN). The order of the tests was counterbalanced across subjects. The CAP battery of tests was performed in a single 1.5-h session with short breaks. Before each test, a subject was familiarized with CAP tests with a training procedure.

The FPT (Pinheiro and Ptacek, 1971) was comprised of 40 binaurally presented at 60-dB HL triplets of 200-ms sine wave tones (180 plateau, rise/decay time of 10 ms) of either a low (880 Hz) or high (1122 Hz) frequency. The task was to verbally report the order of the tones (e.g., /high/–/low/–/high/). Each triplet consisted of 1, 2, or 3 identical sounds (a low or a high tone). An Inter-Tone-Interval (ITI) of 200 ms was utilized. The auditory sequences within the test were presented in a pseudo-random order. The percentages of correct responses were analyzed.

The DPT (Musiek et al., 1990) included 40 binaural 3-element sequences of 1,000-Hz sine wave tones (rise/decay time of 10 ms), differing in duration. The tones were either short (250 ms) or long (500 ms) and subjects were asked to repeat the order of the tones within a sequence (e.g., /short/–/long/–/long/). An ITI of 300 ms was utilized. Stimuli were presented at 60 dB HL. Analogous to the FPT procedure, the percentages of correct responses were calculated.

The GDT (Keith, 2000) measured the shortest length of a silent gap embedded in white noise required for perceiving and reporting the silent gap. The stimulus was a 500-ms white noise presented to both ears at 50 dB HL. An adaptive procedure was applied to search for the length at which there was a 50% chance of detecting the noise with a gap and a 50% chance of detecting a noise without a gap (Leek, 2001). The task consisted of pressing a response key when there was a gap embedded in noise. The minimal gap duration was determined in a 2-stage procedure. In the first stage, stimuli with varying gap durations were presented. The initial gap duration was 10 ms and either decreased or increased by half of its length, depending on the correctness of the subject's responses. This part of the test was continued until a subject failed three times to detect a gap of the same duration. This gap duration was then applied in the primary test and was adjusted in accordance with the individual subject's performance (i.e., it increased by 2 ms following a false alarm, a button pressed in the absence of a noise with a gap, or a miss, no reaction to a gap stimulus, and decreased by 2 ms after a hit, a correct gap detection). The test was terminated after 7 reversals. A reversal was defined as a hit followed by a miss (or a false alarm), or a miss (or false alarm) followed by a hit. The average of the 5 most difficult reversals determined the minimum gap duration.

During the DDT (Musiek, 1983), children were presented with a sequence of 2 different digits in the left ear, while concurrently given a sequence of 2 different digits in the right ear. The task was to repeat the digits from both ears; the digits used were from 1 through 10. There were 40-digit pairs (20 pairs per ear) and auditory stimuli were presented at 60 dB HL. The percentages of correctly reported digits, both separately from the left and right ears, as well as the difference in performance between the right and the left ear in DDT [the right-ear advantage (REA)^1^], were calculated.

In the aSpN test, single-syllable Polish words (Harris et al., 2004) were successively presented against a background of 16-talker babble speech; the task was to repeat the words presented. The words were delivered to both ears with the use of different signal-to-noise ratios (SNRs). An initial (maximum) SNR was 9 dB and the minimum was −15 dB. Negative SNRs indicate that the background noise was louder than the target word and positive values corresponded to when a target word was louder than the background noise. In the aSpN test, an adaptive procedure was applied in which, initially, the SNR decreased by 4 dB after each correct response. From the moment a subject did not respond correctly for the first time, the SNR was increased by 2 dB following each incorrect word and decreased by 2 dB following each correctly repeated word. The aSpN test measures the minimum SNR required to correctly recognize words 50% of the time. The calculations were performed in accordance with the Wilson and McArdle approach (Wilson and Burks, 2005) and were based on the 5 most difficult reversals (i.e., correctly repeated words followed by an incorrect one, or lack of a response, or incorrect answers followed by a correct one). The test was concluded after 7 reversals.

### QEEG data acquisition and analysis

EEG data were acquired using a Mitsar 21 channel EEG system (Mitsar Ltd., St. Petersburg, Russia). Nineteen silver-chloride electrodes were applied in accordance with the International 10–20 standard (Jasper, 1958). The ground electrode was placed on the forehead. All electrode impedances were kept below 5 kOhm. The input signals referenced to the linked ears were filtered between 0.5 and 50 Hz and digitized at a rate of 250 Hz. For each subject, the EEG data was recorded twice: under “Eyes Open” (EO) and “Eyes Closed” (EC) resting conditions. Both “Eyes Open” and “Eyes Closed” blocks of registration last 3 min. If the recorded signal was contaminated with a large number of artifacts (e.g., excessive movements and/or blinking), the session of EEG data acquisition was extended to a maximum of 5 min. To minimalize the number of artifacts, the subjects were asked to keep their neck and facial muscles relaxed and to refrain from making unnecessary eye movements.

QEEG studies were performed individually in a soundproof room. Each subject sat in a comfortable chair with a distance of 1 meter from the screen. During the EO block, the subject was asked to keep the eyes fixated on the black point that was constantly displayed in the center of the screen. In the EC block, the instruction was to relax with eyes closed and not to think about anything special. Both the EO and EC blocks were counterbalanced across the study subjects.

Quantitative data analysis using WinEEG 2.84 software (Mitsar Ltd.) was conducted offline. The weighted average reference montage prior to quantitative data processing was applied (Lemos and Fisch, 1991). Eye-blink artifact's were corrected by zeroing the activation curves of individual ICA components corresponding to eye-blinks (Vigário, 1997; Jung et al., 2000). In addition, epochs with excessive amplitudes (>100 μV) and/or excessive high (>35 μV in the 20–35-Hz band) and slow (>50 μV in the 0–1-Hz band) frequency activities were automatically marked and excluded from further analyses. Finally, EEG was manually inspected to verify if all artifacts were properly removed. The EEG signal period analyzed in each subject was no shorter than 1 min.

Artifact-free, continuous EEG signals were divided into 4.096-s epochs using a Hanning time window (epochs were overlapped by 50%) and submitted to a FFT. Absolute power spectra (a measure of the intensity of energy computed in a series of frequency bands for a discrete time interval) (squared microvolts) (Hughes and John, 1999) for each subject, and separately for each condition, were calculated. Spectra computed with numerous averaged epochs less than 30 were not included in the analysis. The power spectra were also transformed using a natural logarithm for normalization.

The absolute power was determined separately for EEG signals recorded from each electrode and computed for the following frequency bands: delta (1.5–4 Hz), theta (4–8 Hz), alpha (8–12 Hz), low (12–15 Hz), middle (15–18 Hz), and high (18–25 Hz) beta. These ranges overlapped with those used in numerous previous studies (Thatcher, 1998, 2000; Thatcher et al., 2009; Cahn et al., 2010; Engelbregt et al., 2012) and QEEG databases (HBI database; Kropotov, 2009), as well as the Neuroguide database (Thatcher et al., 2003), which allows the comparison of presented results with the outcomes obtained by other authors. The absolute EEG power for particular frequency bands was analyzed separately for the EO and EC states. These conditions have been commonly used in research owing to the simplicity and relative uniformity of the EEG recording procedure. EO and EC recordings can also be compared across laboratories and populations with a relatively high reliability (Thatcher and Lubar, 2009).

### Statistical analysis

The statistical analyses were performed with SPSS 20.0 software (SPSS Inc., Chicago, Illinois). The Shapiro–Wilk's test was run to check for normality of each variable distribution and Levene's test was used to verify whether the variances the analyzed age groups were homogeneous. When a distribution was significantly different from normal and/or the variances were not homogeneous, the results were transformed to make the variances of the different groups equal and/or to normalize the data. When the data still did not meet the assumptions of parametric statistics, non-parametric tests were used. As a result, parametric independent *t*-tests and the non-parametric Mann–Whitney *U*-test were applied for intergroup comparisons of CAP test battery results, respectively. Only the REA index was calculated using the Mann–Whitney *U*-test. Repeated-measures analysis of variance (ANOVA) with electrode (19 levels) and eye condition (EO vs. EC) as repeated-measure factors, and group (CAPD vs. TDC) as an intergroup factor, were conducted separately on the delta (1.5–4 Hz), theta (4–8 Hz), alpha (8–12 Hz), low (12–15 Hz), middle (15–18 Hz), and high (18–25 Hz) beta frequency bands. The results of these analyses were reported with the Greenhouse–Geisser correction. Bonferroni's tests were conducted for *post-hoc* comparisons. In all performed tests, *p* < 0.05 were considered to be statistically significant.

Additionally, to better explore the relations between the CAP battery performance and the QEEG data, Pearson's or Spearman's correlation coefficients (for normal or abnormal data distribution, respectively) between the CAP test results and the absolute power of particular frequency bands have been calculated in all subjects (separately for the EO and EC blocks).

## Results

### Central auditory processes

Table 1 shows the results of CAP tests and some reference values for these tasks. Children with CAPD, relative to TDC, achieved significantly less correct responses in DDT for both right and left ears, as well as in FPT and DPT. Intergroup differences in GDT and aSpN were not significant.

**Table 1:** Reference values for Polish CAP tests developed for typically developing children at the age group corresponding to the age of children recruited to the present study and descriptive statistics (mean values, standard deviations, medians and median ranges) as well as the *t* or *z*-values obtained in the *t*-tests or U Mann-Whitney's tests comparing two experimental groups.

	**Reference data**		**TDC**		**CAPD**		
**CAP**	**$\bar{X\;}$(*SD*)**	**Me**	**$\bar{X\;}$(*SD*)**	**Me (min–max)**	**$\bar{X\;}$(SD)**	**Me (min–max)**	***z/t***
DDT_R (%)	88.6 (7.39)	90.0 (72.5–100)	86.89 (12.65)	91.25 (42–100)	73.70 (11.86)	77.5 (50–90)	3.59^***^
DDT_L (%)	80.7 (7.48)	80.0 (70.0–95.5)	79.40 (11.37)	80.0 (57.5–97.5)	51.85 (20.93)	55.0 (7.5– 90)	5.74^***^
FPT (%)	79.6 (11.82)^a^	79.0 (50–100)^a^	78.94 (14.71)	81.25 (50–100)	32.69 (17.04)	32.5 (0–72.5)	10.31^***^
REA index	0.04 (0.04)	0.03 (−0.04–0.18)	0.04 (0.1)	0.05 (−0.31–0.19)	0.2 (0.25)	0.1 (-0.11– 0.82)	2.13*
DPT (%)	83.4 (9.35)^b^	85.0 (62.5–100)^b^	87.73 (11.31)	90.0 (52.5–100)	45.19 (19.29)	42.5 (17.5–82.5)	8.36^***^
GDT (ms)	2.97 (0.47)	3.0 (2.10–4.0)	4.18 (6.6)	2.65 (1.92–35)	4.28 (4.20)	2.9 (1.3–20.25)	0.55
aSpN (dB)	−1.2 (1.25)	−1.4 (−3.4–2.2)	−0.5 (1.44)	−0.5 (−3–3)	−0.15 (1.79)	0 (−3– 4)	0.77

**p < 0.05,

*** *p < 0.001; TDC, typically developing children; DDT_R, Dichotic Digit Test for the right ear; DDT_L, Dichotic Digit Test for the left ear; REA, Right ear advantage; FPT, Frequency Pattern Test; DPT, Duration Pattern Test; GDT, Gap Detection Test; aSpN, adaptive Speech in Noise*.

a *Reference data from Dajos et al. (2013)*.

b *Reference data from Włodarczyk (2013)*.

*The reference data that has been not published yet was collected from 49 healthy children (25 girls and 24 boys), aged from 10 to 11 years. These data is used in the Institute of Physiology and Pathology of Hearing as the reference for clinical groups*.

### QEEG data

**Figure 1:**
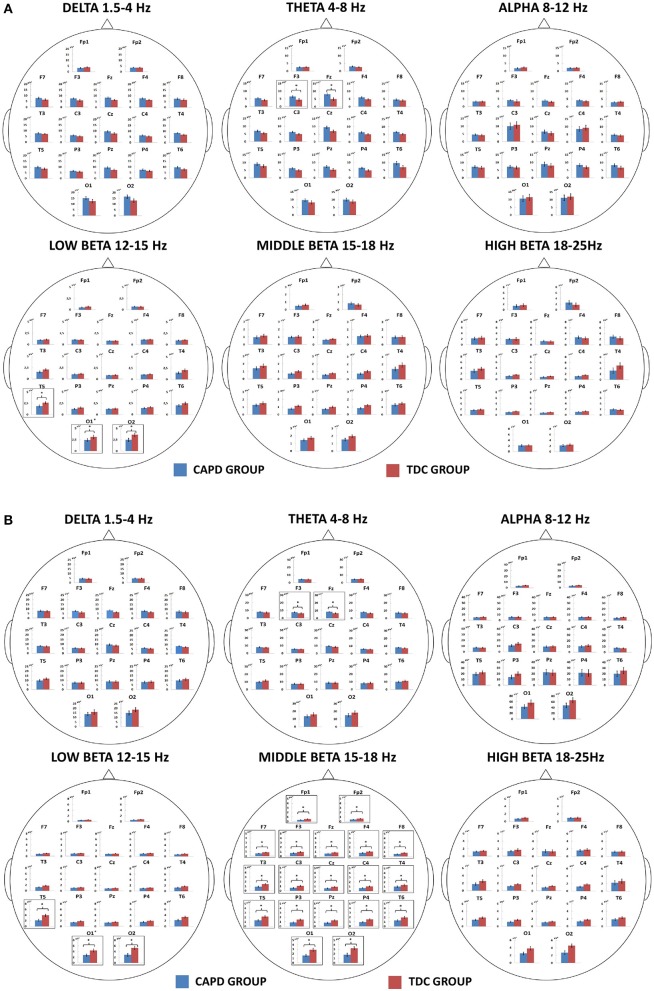
Mean absolute powers for particular frequency bands (delta, theta, alpha, and low, middle, and high beta) calculated from EEG signals recorded in the groups of CAPD and typically developing children (TDC) in “Eyes Open” **(A)** and “Eyes Closed” **(B)** conditions and from electrodes placed on scalp according to a 10–20 standard (Jasper, 1958). The significant differences (*p* < 0.05) in absolute EEG powers between the CAPD and TDC groups at particular electrodes are indicated by asterisks and black frames.

Figure 1 shows the results of intergroup comparisons of the distribution of absolute power in the scalp for particular frequency bands, separately for the EO (Figure 1A) and EC (Figure 1B) conditions.

#### Delta (1.5–4 Hz)

**Figure 2:**
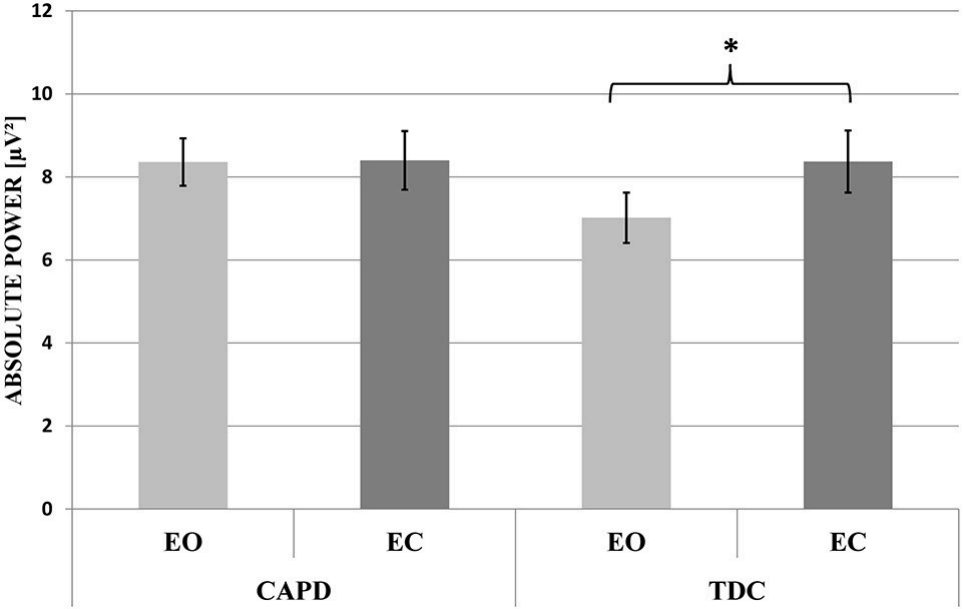
The mean absolute power of the delta frequency band in EEG signals recorded from the CAPD and TDC groups in “Eyes Open” (EO) and “Eyes Closed” (EC) blocks. The significant difference (*p* < 0.05) in the mean delta power between EO and EC conditions in the TDC group is indicated by an asterisk.

The analysis showed a significant main effect of the condition [*F*_(1, 49)_ = 6.65, *p* = 0.013, eta^2^ = 0.12], resulting in greater delta power during the EC ($\bar{X}$ = 8.39 ± 0.51 μV) than in the EO condition ($\bar{X}=7.69\pm$ 0.42 μV), as well as a significant interaction: condition × group [*F*_(1, 49)_ = 5.15, *p* = 0.028, eta^2^ = 0.095]. *Post-hoc* comparisons revealed greater delta power in the EC ($\bar{X}=$ 8.37 ± 0.75 μV) than in the EO condition ($\bar{X}\;$ = 7.02 ± 0.61 μV), but only for the TDC group (Figure 2). The main effect of group membership and other interactions were not significant.

#### Theta (4–8 Hz)

There was a significant main effect of condition [*F*_(1, 49)_ = 47.01, *p* = 0.001, eta^2^ = 0.49], as well as significant interactions: condition × group [*F*_(1, 49)_ = 5.58, *p* = 0.022, eta^2^ = 0.10] and group × electrode [*F*_(1, 49)_ = 2.03, *p* = 0.041, eta^2^ = 0.04]. Theta power was significantly greater in the EC than in the EO condition ($\bar{X}=$ 8.63 ± 0.67 μV and $\bar{X}=$ 6.13 ± 0.39 μV, respectively). *Post-hoc* comparisons for condition × group interaction revealed a tendency (p = 0.058) toward a greater theta power ($\bar{X}=$ 6.88 ± 0.53 μV) in the CAPD group than in the TDC group ($\bar{X}=$ 5.37 ± 0.57 μV), but only for the EO condition. *Post-hoc* comparisons for the group × electrode interaction showed higher theta power in the CAPD group than in the TDC group at F3 ($\bar{X}=$ 6.65 ± 0.52 μV and $\bar{X}=$ 4.97 ± 0.55 μV, respectively) and Fz ($\bar{X}=$ 8.42 ± 0.77 μV and $\bar{X}=$ 5.55 ± 0.82 μV, respectively). Other effects were not significant.

#### Alpha (8–12 Hz)

Repeated-measures ANOVA revealed a significant effect for condition [*F*_(1, 49)_ = 110.02, *p* = 0.001, eta^2^ = 0.69], resulting in a greater alpha power in the EC than in the EO condition ($\bar{X}=$ 15.49 ± 1.40 μV and $\bar{X}=$ 6.15 ± 0.53 μV, respectively). The main effect of “group” and any interaction effects were not significant.

#### Low-frequency beta (12–15 Hz)

The low-frequency beta analyses showed two significant interactions: condition × group [*F*_(1, 49)_ = 4.14, *p* = 0.047, eta^2^ = 0.08] and electrode × group [*F*_(1, 49)_ = 2.77, *p* = 0.05, eta^2^ = 0.05]. Children with CAPD demonstrated reduced low-frequency beta power ($\bar{X}=$ 1.32 ± 0.14 μV) compared to the TDC ($\bar{X}=$ 1.88 ± 0.15 μV), but only for the EC condition. Furthermore, independent of condition, the CAPD group, relative to TDC, had reduced low-frequency beta power at T5 ($\bar{X}=$ 2.03 ± 0.03 μV vs. $\bar{X}=$ 3.30 ± 0.32 μV), O1 ($\bar{X}=$ 2.61 ± 0.34 μV vs. $\bar{X}=$ 3.75 ± 0.37 μV) and O2 ($\bar{X}=$ 2.71 ± 0.43 μV vs. $\bar{X}=$ 4.46 ± 0.45 μV).

#### Middle-frequency beta (15–18 Hz)

The middle-frequency beta analyses revealed a significant interaction: condition × group [*F*_(1, 49)_ = 8.02, *p* = 0.007, eta^2^ = 0.14], showing decreased middle-frequency beta power in children with CAPD ($\bar{X}=$ 0.87 ± 0.11 μV), as compared to TDC ($\bar{X}=$ 1.38 ± 0.12 μV), but only for the EC condition. Other effects were not significant.

#### High-frequency beta (18–25 Hz)

Repeated-measures ANOVA revealed no significant main effects or interactions between the analyzed factors.

### Correlation analysis

**Figure 3:**
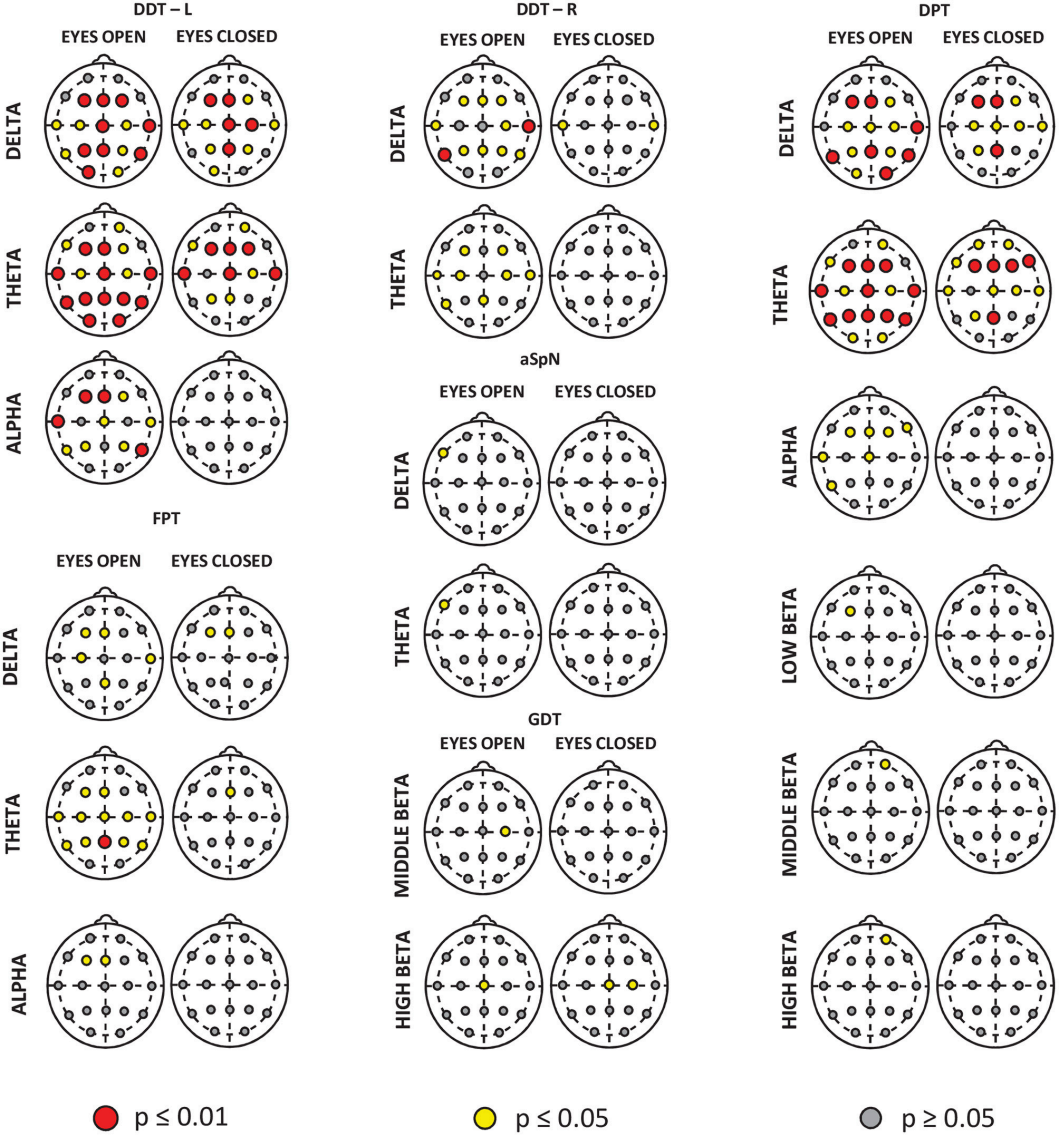
Spatial distributions of correlations between the CAP tests results and mean absolute powers for the individual frequency bands. The correlations were computed in the whole study group (CAPD + TDC), separately in the “Eyes Open” and “Eyes Closed” condition and for signals from each electrode. For better visualization, only the frequency bands for which the absolute power on at least one electrode is significantly related to the CAP test performance are shown. The electrodes with significant correlations are marked with larger red and smaller yellow circles corresponding to the significance level of the correlation coefficients.

**Figure 4:**
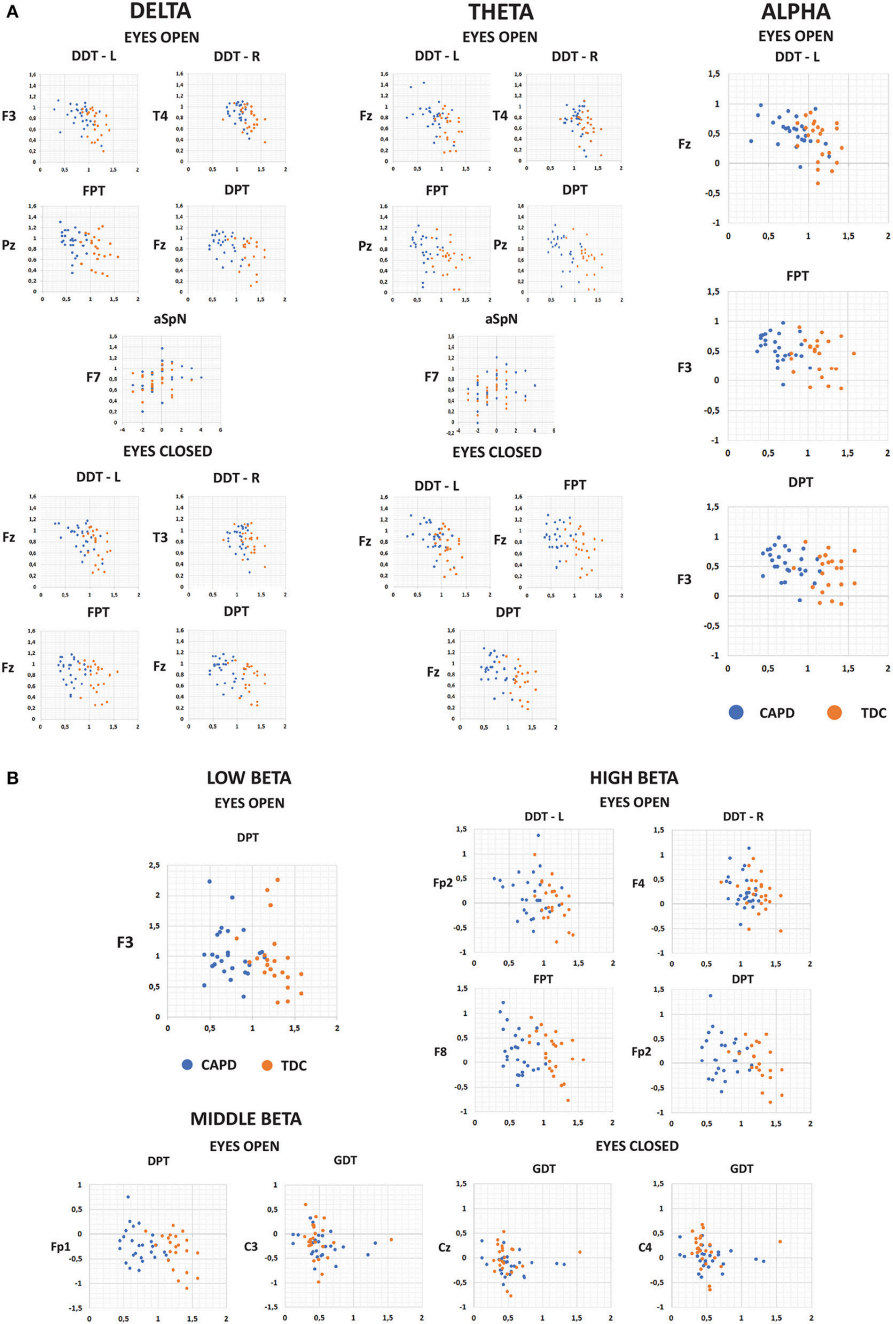
The scatter-plots showing correlations between the normalized scores of CAP tests and normalized values of absolute power of slow **(A)** and fast **(B)** frequency bands in EEG signal recorded under the “Eyes Open” and “Eyes Closed” conditions. Only scatter-plots for electrodes with the highest significant correlations are presented details (see Tables S1, S2).

Detailed results of correlation analysis are presented in Figures 3, 4 and Tables S1, S2.

#### Delta (1.5–4 Hz)

The higher was the delta power in the EO condition at frontal (Fz, F3, F4), central (C4), and temporo-parietal (T3, T4, T5, T6, Pz, P3, P4) areas as well as in the EC block in the temporal region (T3 and T4), the fewer were the correct responses in DDT for right ear. Delta power in the EO block at almost all electrodes (except for Fp1, Fp2, F7 and F8) and in the EC condition at Fz, F3, F4, Cz, C3, C4, T3, T4, Pz, P3, P4, O1 electrodes correlated negatively with the correctness level in DDT for left ear.

The higher was the delta power at Fz, F3, C3, T4, and Pz electrodes in the EO block and at Fz, F3 during the EC block, the fewer were the correct responses in FPT.

Furthermore, delta power in the EO block at the frontal (Fz, F3, F4), central (Cz, C3, C4), temporal (T4, T5, T6), parietal (Pz, P3, P4), and occipital (O1, O2) areas as well as in the EC condition at Fz, F3, F4, Cz, C3, C4, T4, Pz, and P3 electrodes correlated negatively with DPT correctness level.

In the EO block an increase of delta power at F7 electrode in relation to increasing SNR in aSpN test was found.

No significant associations of delta power with aSpN results in the EC block and with GDT measures in both resting-state conditions were found.

#### Theta (4–8 Hz)

In the EO condition, theta power at electrodes located over the frontal (F3, F4), central (C3, C4), temporal (T3, T4, T5), and parietal (Pz) areas correlated negatively with the percent of correct responses in DDT for right ear. No significant associations between theta power during the EC block and the results in DDT for right ear were observed. Furthermore, the higher was the theta power at almost all electrodes except for Fp1 and F8 in the EO condition and at frontal (Fp2, Fz, F3, F4, F7), central (Cz, C4), temporal (T3, T4), and parietal (Pz, P3) electrodes in the EC block, the fewer were the correct responses in DDT for left ear.

Theta power at frontal (Fz, F3), central (Cz, C3, C4), temporal (T3, T4, T5, T6), and parietal (Pz, P3, P4) regions in the EO block and at Fz electrode during the EC block correlated negatively with FPT scores.

Theta power in the EO condition at almost all electrodes except for Fp1 and F8 was negatively related to DPT correctness level. Furthermore, the greater was the theta power in the frontal (Fp1, Fp2, Fz, F3, F4, F7 and F8), central (Cz, C4), temporal (T3, T4), and parietal (Pz, P3) areas in the EC block, the fewer were the correct responses in DPT.

In the EO condition theta power at F7 electrode correlated positively with SNR values in aSpN test, whereas no substantial relations of theta power with aSpN measures were found during the EC block.

Theta power also did not correlate significantly with GDT results.

#### Alpha (8–12 Hz)

Significant correlations between alpha power and CAP tests performance were found only for the EO condition. Specifically, the higher was the alpha power at Fz, F3, F4, Cz, T3, T4, T5, T6, Pz, P3, and P4 electrodes, the fewer were the correct responses in DDT for left ear.

Alpha power at frontal area (Fz and F3 electrodes) correlated negatively with FPT results.

There were also negative relationships between alpha power at Fz, F3, F4, F8, Cz, T3, and T5 electrodes and DPT correctness level.

Alpha power had no significant association with DDT for right ear, aSpN and GDT measures.

#### Low-frequency beta (12–15 Hz)

The only significant relation here was between low beta power at F3 and Pz electrodes during the EO block and DPT scores (i.e., the higher low beta power, the fewer correct responses in DPT).

Low beta power did not substantially correlate with other CAP tests performance.

#### Middle-frequency beta (15–18 Hz)

Again, the DPT correctness level decreased with increasing middle beta power at Fp2 electrode only during the EO block.

Furthermore, middle-frequency beta power at C3 and C4 electrodes correlated negatively with GDT results. Specifically, the higher was the beta power the shorter was a minimum gap which could be detected in noise.

No significant correlations were found between the middle-frequency beta power and any other CAP tests measures.

#### High-frequency beta (18–25 Hz)

There are significant negative correlations between the high-frequency beta power at F4 during the EO block and DDT scores in right ear.

Furthermore, the greater was the high-frequency beta power at Fp2 electrode in the EO condition, the fewer were the correct responses in DDT for left ear, FPT and DPT. In the EO block, high-frequency beta power at F8 electrode was negatively related to FPT and DPT correctness level.

The greater high-frequency beta power at Cz (the EO condition), the shorter was the detectable gap in noise. For the EC block, beta power at Cz and C4 correlated negatively with GDT results; this frequency band power at O1 electrode was positively associated with the percentage of correct responses in FPT.

## Discussion

To our knowledge, this is the first study demonstrating an abnormal QEEG pattern in children with CAPD profiles characterized by poor auditory pattern recognition (temporal patterning) and dichotic listening (Figures 1, 2 and Table 1).

### CAPD profile

According to current guidelines of the American Academy of Audiology (American Academy of Audiology, 2010) and the British Society of Audiology (British Society of Audiology, 2011a), CAPD is present when at least 2 CAPs are disturbed. Additionally, such impairments should be manifested by poor performance on both verbal and non-verbal CAP tests. Children who participated in our study showed deficits in both non-verbal sequencing tests (FPT and DPT) and verbal binaural integration/separation task (DDT). This indicates that the diagnostic criteria of having at least 2 CAP impairments, one of which is verbal and the other is non-verbal, were fulfilled.

Children with CAPD suffer from different auditory deficits that may form distinct subprofiles. Bellis and Ferre (1999) distinguished three main auditory processing subtypes: Auditory Decoding Deficit, Prosodic Deficit, and Integration Deficit, which represent dysfunctions in the auditory cortex of the left hemisphere, right hemisphere, and interhemispheric connections (corpus callosum), respectively. In our study, children with CAPD displayed impaired temporal patterning, left ear deficit in the dichotic listening test and normal low-redundancy speech tests. These symptoms are characteristic for the Prosodic Deficit (Bellis, 1999, 2003). However, the auditory profile presented in this work also included lowered scores in DDT in both ears suggesting the presence of Auditory Decoding Deficit. Finally, the left ear deficit was accompanied in our study by disturbed temporal patterning in the linguistic labeling condition (i.e., subjects were asked to orally report the order of sounds in a sequence), which may be indicative of the Integrative Deficit as well. In this CAPD subtype, performance of temporal patterning tasks would be better if subjects were asked to hum the melody instead of repeating verbally the names of consecutive auditory stimuli. Deficits observed in the linguistic labeling condition may only indicate that the information transfer to the left hemisphere is affected, while the perception of acoustic contour is preserved (Musiek et al., 1980). Since in our study we use only one kind of response in both applied temporal pattering tasks (FPT and DPT), we cannot conclude the presence or absence of symptoms characteristic for the Integration Deficit based on these tests results (Bellis, 1999). As Bellis (2003) has observed, these three auditory subprofiles may exist singularly or in combination. In our study left- and right-hemisphere-based CAPD co-occurred with the interhemispheric-based deficit. That could as well indicate a severe auditory problem or a global cognitive (attention and/or short-term memory) impairment not limited to the central auditory system.

The vast majority of children with CAPD included in our study displayed a configuration of CAP tests results corresponding to the Prosodic or Integration Deficit. Only a few children showed, besides an impaired auditory pattern recognition and dichotic listening tasks, also an elevated gap detection threshold (the minimum gap duration that a listener can detect) in GDT and increased signal-to-noise ratio in aSpN test. The performance of these few children did not significantly affect the outcomes of the whole CAPD group.

An important temporal aspect of audition, which is often tested as a part of a CAPD diagnosis, is temporal resolution (i.e., an ability of the auditory system to respond to rapid changes in sounds). In the present work, both children with CAPD and without auditory processing deficits obtained a minimum gap duration of about 4 ms, which is a comparable value with those reported by other authors in healthy controls (Musiek et al., 2005; Shinn et al., 2009; Amaral and Colella-Santos, 2010; Perez and Pereira, 2010; Zaidan and Baran, 2013). However, the exact comparison of our GDT results with previous findings is not possible owing to different experimental procedures and/or methods of establishing the threshold value that have been used in other studies. Temporal resolution is an important aspect of speech perception and it has been well-documented that the minimum gap duration is higher in language-disordered populations, such as those with dyslexia or SLI (Sharma et al., 2009; Zaidan and Baran, 2013). In our study, children with CAPD did not exhibit any concomitant reading or writing problems; therefore, abnormal temporal resolution was not necessarily expected in this study population.

Children with listening difficulties showed deficits in FPT and DPT, which required an ability to reproduce 3-tone sequences (Table 1). Normal gap detection ability, combined with impaired auditory pattern recognition, in children with CAPD may indicate attention and/or short-term memory problems rather that temporal processing deficits. Since temporal sequencing is crucial for the perception of speech, music, and prosody (i.e., rhymes, accent or intonation, or the emotional state of a speaker) (Musiek et al., 1980; Musiek and Chermak, 1995; Bellis, 2003), children who experience difficulties in performing FPT and DPT may suffer from more general perceptual deficits.

In FPTs and DPTs, a child is asked to focus on an auditory pattern, retain the pattern in short-term memory, and then repeat the sequence. Therefore, one cannot exclude the situation of when a child fails to perform the task owing to increased fatigue and/or distractibility. Other authors had similar conclusions and even proposed to include cognitive tests into CAPD assessments to clarify the extent of attention and short-term memory affecting CAP test performance (Sharma et al., 2006, 2009; Ahmmed et al., 2014). Assessing children with reading disabilities demonstrated that auditory attention and memory accounted for more than 20% of the variance in FPT scores. To summarize, poor performance on FPTs and DPTs may arise from a temporal deficit and a more general deficit in attention/short-term memory.

Tonal pattern recognition typically involves both hemispheres (specifically the temporal lobe) as well as interhemispheric connections. In the central auditory system both ipsi- and contra-lateral processing pathways are much more symmetric than for other sensory modalities (Rauschecker and Scott, 2009). Especially the ipsi-lateral connections may more significantly affect auditory processing than previously thought (Schneider et al., 2009). Thus, tonal pattern recognition deficit may result from impaired interactions of ipsi-, contra-lateral and inter-hemispheric processes.

In the present study, children with CAPD displayed difficulties when performing DDTs for both the right and left ear (Table 1). Since DDT measures binaural integration and separation, which is necessary for the management of auditory information presented simultaneously to both ears (Bellis, 2003), poor results on this test could indicate an impairment of this function. In a typical dichotic listening task, speech sounds (e.g., digits) exposed to the right ear directly reaches the language centers of the left hemisphere, whereas the auditory input to the left ear is initially transferred to the right hemisphere, and then through the corpus callosum, reaches the speech areas of the left hemisphere (Kimura, 1961a,b; Bellis, 2003). Therefore, poor DDT performance in both ears could result from a dysfunction in both hemispheres, or in only one hemisphere (e.g., the left) when it is accompanied by involvement of corpus callosum. In the latter case, lowered scores in the left ear are caused by an engagement of callosal fibers and the right ear deficit is observed owing to a contralateral ear effect. Musiek and Pinheiro (1987) reported that reduced performance in both ears could also result from brainstem lesions.

Both the CAPD and control groups obtained higher percentages of correct responses in the right than left ear (Table 1), which indicates the typical right ear (left hemisphere) advantage (REA) effect for the processing of dichotomously presented verbal sounds (Kimura, 1961a; Bryden, 1963; Satz et al., 1965; Bellis, 2003). Furthermore, children with CAPD demonstrated a higher REA index than their healthy peers, which is concurrent with a previous finding regarding a larger REA in dichotic listening tasks of children with CAPD compared to TDC (Bellis et al., 2011). This effect combined with considerably lower scores in the left ear may be caused by the immaturity of the right hemisphere and/or overcompensation of the left hemisphere. However, since children with CAPD showed a bilateral decrease in DDT performance, it is more likely that other factors related to attention and short-term memory may affect the outcomes. In this case, lowered DDT scores could result from increased arousal and/or distractibility during the experimental procedure, which requires dividing attention between the two ears and making decisions about what is heard.

Finally, children with CAPD were not significantly different from TDC in terms of performance on the aSpN test, for subjects in the present study (Table 1). These outcomes could be interpreted as demonstrating the normal ability of CAPD children to perceive speech in challenging listening conditions. Nilsson et al. (1994) suggest that aSpN evaluates phonetic decoding, which is an ability to use external information (extrinsic redundancy) or previous auditory experiences (intrinsic redundancy) to complete distorted acoustic signal and recognize it correctly (Masquelier, 2002). In our study, children with listening difficulties did not show deficits in this test, which indicates normal phonetic decoding in this CAPD subtype.

In the following paragraphs, we will refer to our QEEG outcomes in terms of other neurodevelopmental disorders in an effort to delineate the changes in bioelectrical resting-state activity that may be specific to a core auditory deficit.

### Summary of QEEG results

In children with CAPD, we found alternations in delta and theta, as well as low- and middle-frequency beta bands (Figure 1). Specifically, in children with listening difficulties there was no effect of greater delta power under the EC condition compared to the EO condition that was observed in the healthy control group (Figure 2). Additionally, CAPD, relative to TDC, showed a tendency toward increased theta power in the EO state and, irrespective of the condition, enhanced theta power in the anterior brain area, at the F3 and Fz electrodes. Intergroup differences were also found for low- and middle-frequency beta power. Specifically, children with CAPD showed reduced low-frequency beta power, with this effect observed in both resting-state conditions in the posterior brain area (T5, O1, and O2 electrodes). Considering middle-frequency beta power, there was a decreased power in the CAPD group compared to the control group, but only under the EC condition.

### CAPD profile and resting-state QEEG pattern

Recently, imaging techniques have gained interest as potential methods for CAPD evaluation (Micallef, 2016). Investigating brain activity for clinical purposes is often associated with a dilemma of what paradigm should be applied. Firstly, patients use different cognitive strategies while performing a cognitive task, which substantially increases the variability of results. Secondly, a task-related paradigm induces only specific activations and not the whole brain network. These limitations may be overcome by using a resting-state protocol in which there are no external tasks. This paradigm allows the examination of the whole brain without restrictions to the areas involved in a particular task. Another advantage of this protocol is its short duration (few minutes), which is advantageous for testing children who have difficulty being motionless and sustaining attention for a long time.

Resting-state QEEG has been proven to be highly reliable and reproducible (Salinsky et al., 1991; Burgess and Gruzelier, 1993; Corsi-Cabrera et al., 1997; Hughes and John, 1999; Thatcher and Lubar, 2009). In many developmental disorders including ADHD (Clarke et al., 1998, 2001a,b; Monastra et al., 1999, 2001; see Barry et al., 2003 for a review; Arns et al., 2008), dyslexia (Duffy et al., 1980; Arns et al., 2007), or autism (Pop-Jordanova et al., 2010; Linden and Gunkelman, 2013 for a review) different abnormal bioelectrical activity patterns have been reported. These outcomes indicate that QEEG may be an adequate tool for investigating EEG activity, specific to a core auditory deficit in children with listening difficulties.

In contrast with TDC, the CAPD group showed no effect of reduced delta power in the EO compared to the EC state (Figure 2). After visual inspection of Figure 2, one may claim that a lack of difference between these two resting conditions was owing to increased delta power during the EO block in the children with CAPD. Delta rhythm may have both thalamic and cortico-thalamic origins (Steriade, 2006). These structures are considered to be involved in maintaining cortical arousal (the current energetic level of the organism), and/or activation, which reflects the task-related mobilization of energy needed for task performance (Cannon, 2012). Therefore, we hypothesized that an increased resting delta power in children with CAPD reflects abnormalities in the excitation levels, which, in turn, may contribute to auditory processing and attention deficits. Enhanced delta power in children with CAPD in the EO state may reflect diminished arousal and a reduced readiness to respond to stimuli.

Delta rhythm in a normal awake state is observed occasionally and becomes prominent only during drowsiness (Britton et al., 2016). It is also visible in the wakefulness of patients with brain injuries who suffer from slowing of reactions and increased distractibility (Gotman et al., 1973; Jackel and Harner, 1989; Logar and Boswell, 1991; Harmony et al., 1995; Murri et al., 1998; Fernández-Bouzas et al., 1999, 2002; Lukashevich et al., 1999; Babiloni et al., 2006).

Increased delta power has previously been observed in individuals with ADHD, mainly in the posterior (Matousek et al., 1984; Clarke et al., 1998, 2001a; Koehler et al., 2009), frontal, and fronto-central regions of the brain (Matsuura et al., 1993; Koehler et al., 2009; Kropotov, 2009). Children with dyslexia also demonstrated slow activity (delta and theta) in the frontal and temporal regions of the brain (Arns et al., 2007). In the children with CAPD in our study, the effect of increased delta power was less focal and generally distributed in the scalp. Further studies are warranted to determine whether resting-state delta rhythm may be useful in discriminating between listening difficulties and other disorders with overlapping symptoms.

In the present study, children with CAPD showed a tendency toward increased theta power during the EO state. Simultaneously, enhanced theta power was observed in the frontal areas, at the F3 and Fz electrodes, irrespective of the resting condition (Figures 1A,B). Similarly to delta rhythm, enhanced theta frequency band power normally occurs with drowsiness and a subconscious mental state (Sih and Tang, 2013). It is also considered a marker of brain pathology (Montgomery et al., 1991; Coutin-Churchman et al., 2003). Increased theta power has been observed in traumatic brain injuries (McClelland et al., 1994; Fenton, 1996), epilepsy (Clemens, 2004), schizophrenia (Clementz et al., 1994; Sponheim et al., 2000; Wichniak et al., 2015), or polysubstance abuse (Coutin-Churchman et al., 2003).

Elevated theta power has been frequently observed in children and adults with ADD/ADHD, both in the EO and EC states (Janzen et al., 1995; Clarke et al., 1998, 2001a,b, 2002; Lazzaro et al., 1998, 1999; Bresnahan et al., 1999; Barry et al., 2002, 2003; Bresnahan and Barry, 2002; Loo and Makeig, 2012), as well as in dyslexia (Arns et al., 2007) and high-functioning autism (Yeung et al., 2016). This effect, found especially in the frontal area of the brain, may reflect poor concentration (Mann et al., 1992) and executive function deficits, such as difficulty in inhibiting inadequate reactions (Pliszka et al., 1996). Similar to individuals with ADHD, children with CAPD in our study showed increased theta power in the anterior region of the brain.

Children with CAPD showed enhanced theta power, accompanied by a decrease of middle-frequency beta power (15–18 Hz), but only during the EC condition in this study (Figure 1B). The effect of increased theta power accompanied by a reduced beta rhythm, also described as an increased theta/beta ratio, is typically observed in subjects with ADHD (Lubar, 1991; Mann et al., 1992; Barry et al., 2003, 2009). More specifically, there is decreased beta power in the parietal area accompanied by elevated frontal theta power (Janzen et al., 1995; Chabot and Serfontein, 1996; Clarke et al., 1998, 2001a; Lazzaro et al., 1999; Monastra et al., 1999; Chabot et al., 2001). An increased theta/beta ratio is thought to represent hypoarousal (Satterfield and Cantwell, 1974; Lubar, 1991; Loo and Barkley, 2005) or task-related underactivation (Barry et al., 2009), which is responsible for an impaired capacity for attentional tasks.

The effect of increased theta and decreased beta power may also account for the maturational-lag hypothesis (Kinsbourne, 1973; Clarke et al., 1998; Lazzaro et al., 1998, 1999). According to this theory, the typical CNS maturation processes' slow rhythms are replaced with fast bioelectrical activity. Therefore, the increased slow rhythms (theta) and decreased fast activity (beta) in our study may reflect underdevelopment of the CNS in children with CAPD, affecting both auditory processing and attentional performance.

Middle-frequency beta power has been considered to reflect the activity of the noradrenergic network, which is involved in sustained attention and working memory processes (Posner and Petersen, 1990; Posner and Raichle, 1994). Previous studies have shown that an enhancement of in the 15–18-Hz band after neurofeedback training was associated with better sustained attention (Lubar and Lubar, 1984; Lubar et al., 1995; Linden et al., 1996). Other studies reported a greater number of false alarms and shorter reaction times in the continuous performance task (Egner and Gruzelier, 2001), or faster, but not necessarily correct responses, in sustained attention tasks (Egner and Gruzelier, 2004). In both these studies, elevated beta power was also accompanied by increased P300 amplitude in an auditory oddball paradigm. The elevated P300 amplitude may have arisen from higher cortical excitation (Polich and Kok, 1995) and improved cognitive processes involved in the stimulus evaluation and the updating of information in the working memory (Donchin and Coles, 1988).

In the present study, children with CAPD who showed decreased power in the 15–18-Hz rhythm. Middle-frequency beta rhythm is also believed to reflect the local inhibition processes in the cortex (Kropotov, 2009), which are carried out by neural networks consisting of inhibitory interneurons (Pfurtscheller et al., 1997). Excitation and inhibition processes in the cortex are in constant competition. The neural network responsible for cortical activation receives and processes stimuli, whereas the inhibitory network terminates the activity after incoming information has been already processed (Kropotov, 2009). Decreased beta power, which was observed in the CAPD subjects in our study, may cause dysfunction of inhibitory processes contributing to difficulty in refraining from inadequate behavioral reactions.

Beta power is also considered to be related to motor and/or sensorimotor functions (Pfurtscheller et al., 1996). This rhythm is particularly evident during constant muscle contraction and suppressed by volitional movements (Baker, 2007), or the image of movement (de Lange et al., 2008). It has been found that beta power in the frontal region increases during inhibition of inadequate motor responses (*go/no go* tasks), whereas beta in the motor cortex decreases in “stop” trials (Swann et al., 2009). Beta power generated in the central sensorimotor areas appears to be a marker of processes that maintain movement representation and block any new movements in the cortex (Engel and Fries, 2010). Therefore, abnormal beta rhythms in the sensorimotor area may reflect impaired periodic recalibration of the sensorimotor system.

In the current study, we observed decreased middle-frequency beta power in the central brain areas in children with CAPD (Figure 1B). This may suggest hyperactivity or hypersensitivity in this group. The 15–18 Hz rhythm was also decreased in the posterior regions of the brain (Figure 1B), which are involved in the modulation of top-down processes (Posner and Dehaene, 1994; Mirsky, 1996; Levy and Swanson, 2001). It has been also found that the central (premotor) parietal network is engaged when attention is directed to visual, auditory stimuli, or both these modalities simultaneously (Saito et al., 2005). Reduced beta activity in this network may reflect attentional deficits.

In the present study, children with CAPD also showed reduced low-frequency beta power (12–15 Hz) in the posterior brain area, under both the EO and EC conditions (Figures 1A,B). Low-frequency beta power in the sensorimotor cortex is called sensorimotor rhythm (SMR), which has been often been described as reflecting cortical inhibition processes (Howe and Sterman, 1972). Neurofeedback training that aimed to increase SMR has been used to suppress activity of the sensorimotor area, or more generally, cortical hyperactivity in children with ADHD (Lubar and Shouse, 1976; Shouse and Lubar, 1979). It has been found that enhancement of SMR coexisted with better results on continuous performance and sustained attention tasks (Egner and Gruzelier, 2001, 2004), or with a clear improvement in semantic working memory, but only limited improvement in attentional processing (Vernon et al., 2003). Furthermore, SMR positively correlated with the amplitude of the P300 component (Egner and Gruzelier, 2001) that is thought to be an index of attention resources allocated to a given task (Polich, 2007).

In the current study, intergroup differences in low-frequency beta power were observed in spatial locations atypical for SMR. More specifically, children with listening difficulties demonstrated decreased 12–15 Hz power in the left temporo-occipital and bilateral occipital areas (Figures 1A,B). Similar to how the temporal lobe processes auditory inputs, the occipital lobe is important for correctly understanding visual information. Therefore, abnormal changes in low-frequency beta power in these regions may indicate problems with processing acoustic and visual stimuli. Further studies, are needed to clarify a relationship between low–frequency beta rhythm and CAPD.

### Relationships between QEEG and CAP tests outcomes

The results of correlation analysis between QEEG and CAP tests outcomes clearly support a link between resting bioelectrical activity and auditory information processing.

In the present study, delta and theta power was negatively associated with DDT for both ears, FPT and DPT results (Figure 4A and Tables S1, S2). The effect of increasing delta and theta power with decreasing correctness level in these tests was observed at most electrodes in both resting conditions, especially in the EO block (Figure 3 and Table S1). Thus, worse performance on temporal patterning and dichotic listening tasks was associated with a distinct delta and theta frequency band power.

Enhanced delta is thought to reflect reduced excitation level (Cannon, 2012) that may contribute to increased distractibility affecting the CAP tests execution. Similarly, increased theta may be related to poor concentration and executive functions deficits (Pliszka et al., 1996). To perform DDT, FPT, or DPT correctly, attention and short-term memory must be involved. Thus, correlations of delta and theta power with the outcomes of above-mentioned CAP tests may be a further evidence supporting a link between enhanced delta and theta rhythms and inattention.

In the present study we did not find any significant differences between CAPD children and TDC in alpha power (Figure 1). However, there were negative correlations between alpha power and the DDT for left ear, FPT and DPT correctness level (the higher alpha power, the fewer correct responses in these tests) (Figure 4A and Tables S1, S2). The most pronounced effect was observed in DDT for left ear in the EO condition (Figure 3 and Table S1). Alpha rhythm is thought to reflect top-down control: it regulates the inhibition of masker (irrelevant) information during speech processing in challenging listening conditions (Strauß et al., 2014). Children with CAPD in our study performed poorly in auditory tests that require processing of verbal sounds presented simultaneously (dichotic listening task). This effect co-occurs with enhanced alpha power. Moreover, in CAPD children increased alpha power coexisted with worse performance of dichotic speech listening task, but only for left ear (right hemisphere) (Figure 4A). Increased alpha (generally or only in the right hemisphere) may reflect over-suppression of the acoustic information presented to the left ear (the right hemisphere).

Significant negative correlations of alpha power with auditory pattern recognition (FPT and DPT tests) results have been also observed. Enhanced alpha band activity has been related to tonic alertness (Dockree et al., 2007) which is thought to increase during internally oriented attention (Verbeke et al., 2014). Enhanced concentration on an internal state is often observed in highly anxious persons. Thus, it is possible that in our study the increased alpha in children with CAPD reflected higher level of anxiety in this group, which might have affected not only the temporal patterning tests performance but also other tasks (DDT for left ear) in which CAPD children achieved significantly worse scores compared to the TDC group.

We also found significant negative associations between the beta band power in the frontal area under the EO condition and the results of temporal patterning tests (DPT) (Figures 3, 4B and Table S1). Since the anterior brain regions are typically engaged in attention and short-term memory (e.g., Buckner, 2004), this relationship may be a further evidence of a substantial contribution of these processes to the auditory performance.

In our study we have also found significant correlations between middle- and high-frequency beta at central electrodes and gap detection threshold in GDT (Figures 3, 4B and Tables S1, S2). In general, fast rhythms (e.g., beta) reflect sampling rate, i.e., adaptation (efficient synchronization) of neural networks to incoming sensory information (Baltus and Herrmann, 2016). The lower power of beta frequency band may reflect disturbed sampling rate (poor temporal resolution) which may result in an elevated gap detection threshold.

### Study limitations and future directions

A major limitation of the current study findings was the small sample size, which prohibits making any strong conclusions based on the obtained results. However, to our knowledge, this is the first study showing preliminary data on the resting-state bioelectrical activity and coexisting attentional deficits in children with listening difficulties. Furthermore, we aim to present the results from larger cohort in the future. We also intend to distinguish CAPD subtypes based on our behavioral and EEG data, which may allow us to design a neurofeedback therapy especially dedicated to particular groups of children with listening problems. A larger sample size will also allow for more advanced analyses of EEG data (e.g., EEG signal coherence), with the examination of any correlation between behavioral and electrophysiological results. We also intend to compare the results in children with both listening difficulties and ADD/ADHD, since these disorders are heterogeneous and characterized by overlapping symptoms, particularly attention deficits, which could affect performance on CAP tests. We believe this would be useful in clinical practice since ADD/ADHD appears to be a potential confounding factor in CAPD evaluation.

## Conclusions

This study present the preliminary electrophysiological results in children with a CAPD subtype characterized by deficits in auditory processing of competing acoustic signals and auditory pattern recognition (or temporal patterning). Changes in the absolute delta, theta, low-, and middle-frequency beta power, may distinguish CAPD from normally developing children. Therefore, QEEG seems a useful tool for improving CAPD evaluation. A potential application of this method to discriminate between different CAPD subtypes and other neurodevelopmental disorders with overlapping symptoms may be an important topic of future research.

We also found the evidence of the relationship between the individual frequency bands in QEEG data and CAPs. Further studies on a larger sample are needed to investigate the clinical relevance of combining resting state electrophysiological data with central auditory processing tests' results.

## Author contributions

RM and ML took part in designing the research, data analysis, and manuscript preparation; MG collected electrophysiological and behavioral data as well as provided the interpretation of their results; EW helped with subject recruitment for the study as well as provided the interpretation of the behavioral data; DG collected behavioral data and provided the interpretation of their results; HS provided valuable comments and remarks on the manuscript and supervised the interpretation of the results. All authors read and approved the final manuscript.

### Conflict of interest statement

The authors declare that the research was conducted in the absence of any commercial or financial relationships that could be construed as a potential conflict of interest.

## Footnotes

^1^REA $=\;\frac{\%\;of\;correct\;responses\;in\;the\;right\;ear\;-\;\%\;of\;correct\;responses\;in\;the\;left\;ear}{\%\;of\;correct\;responses\;in\;the\;right\;ear\;+\;\%\;of\;correct\;responses\;in\;the\;left\;ear}$
