# Supplementary material for: Abnormal Resting-State Quantitative Electroencephalogram in Children With Central Auditory Processing Disorder: A Pilot Study
Source: Front Neurosci. 2018 May 11;12:292. doi: 10.3389/fnins.2018.00292 (PMC5958225; doi:10.3389/fnins.2018.00292)
Supplement: Supplementary file 2 [file Table_2.pdf]

Table S2. The Pearson's or Spearman's correlation coefficients between the mean absolute power calculated separately at each electrode or individual frequency bands and CAP tests results during the "Eyes Closed" condition. Significant correlation coefficients are written in bold ($p < 0.01$) or both in bold and italics ($p < 0.05$).

CAP TEST	Fp1	Fp2	F7	F3	Fz	F4	F8	C3	Cz	C4	T3	T4	T5	T6	P3	Pz	P4	O1	O2
	**DELTA**																		
**DDT_R**	0,033	-0,090	-0,089	-0,221	-0,262	-0,207	-0,090	-0,154	-0,170	-0,253	***-0,280***	***-0,276***	-0,186	-0,166	-0,135	-0,172	-0,192	-0,011	-0,050
**DDT_L**	-0,133	-0,178	-0,133	**-0,416**	**-0,477**	***-0,355***	-0,073	***-0,287***	**-0,406**	**-0,371**	***-0,339***	***-0,319***	-0,183	-0,244	***-0,292***	**-0,370**	***-0,290***	***-0,294***	-0,217
**FPT**	-0,120	-0,154	-0,041	***-0,281***	***-0,319***	-0,207	-0,100	-0,205	-0,206	-0,245	-0,204	-0,220	-0,070	-0,061	-0,147	-0,212	-0,136	-0,021	-0,065
**DPT**	-0,206	-0,239	-0,162	**-0,389**	**-0,44**	***-0,334***	-0,059	***-0,294***	***-0,313***	***-0,312***	-0,275	***-0,319***	-0,143	-0,181	***-0,291***	**-0,350**	-0,271	-0,092	-0,135
**aSpN**	0,116	0,243	0,213	0,157	0,029	0,077	-0,018	-0,026	0,040	0,003	0,200	0,163	0,220	0,216	0,094	0,070	0,026	0,119	0,117
**GDT**	0,074	0,072	-0,163	-0,133	-0,165	-0,153	-0,116	-0,173	-0,129	-0,073	-0,036	0,020	0,040	-0,056	-0,006	-0,080	-0,136	-0,131	-0,126
	**THETA**																		
**DDT_R**	0,011	-0,052	-0,091	-0,204	-0,236	-0,202	-0,078	-0,124	-0,169	-0,191	-0,172	-0,138	-0,055	-0,052	-0,049	-0,064	-0,015	0,078	0,084
**DDT_L**	-0,254	***-0,308***	***-0,303***	**-0,413**	**-0,508**	**-0,379**	-0,194	-0,242	**-0,380**	***-0,296***	**-0,390**	**-0,385**	-0,187	-0,251	***-0,279***	***-0,306***	-0,213	-0,228	-0,167
**FPT**	-0,167	-0,207	-0,137	-0,265	***-0,310***	-0,234	-0,096	-0,170	-0,199	-0,230	-0,247	-0,214	-0,061	-0,071	-0,150	-0,203	-0,115	0,008	0,001
**DPT**	***-0,322***	***-0,356***	***-0,320***	**-0,411**	**-0,486**	**-0,408**	**-0,155**	-0,279	***-0,351***	***-0,339***	***-0,363***	***-0,352***	-0,203	-0,229	***-0,317***	**-0,387**	-0,275	-0,108	-0,094
**aSpN**	0,137	0,242	0,220	0,131	0,060	0,090	0,032	0,034	0,045	0,055	0,138	0,153	0,121	0,102	0,079	0,034	0,036	0,098	0,106
**GDT**	0,003	0,057	-0,120	-0,097	-0,127	-0,054	-0,049	-0,139	-0,094	0,006	-0,035	0,093	0,054	0,134	0,036	-0,062	0,021	0,003	-0,044
	**ALPHA**																		
**DDT_R**	0,044	0,012	-0,105	-0,109	-0,118	-0,113	-0,022	-0,107	-0,076	-0,120	-0,117	-0,093	0,057	-0,087	-0,046	-0,050	-0,120	0,053	0,124
**DDT_L**	0,014	-0,037	-0,119	-0,234	-0,230	-0,150	-0,046	-0,830	-0,207	-0,094	-0,253	-0,266	-0,040	-0,150	-0,143	-0,084	-0,107	-0,030	0,078
**FPT**	0,145	0,086	0,102	0,004	-0,025	-0,020	0,122	-0,001	-0,016	-0,029	-0,040	-0,072	0,096	0,044	0,075	0,020	0,074	0,147	0,156
**DPT**	0,05	-0,018	-0,052	-0,136	-0,155	-0,111	0,014	-0,066	-0,168	-0,133	-0,200	-0,229	0,012	-0,076	-0,059	-0,151	-0,099	0,092	0,129
**aSpN**	-0,044	0,041	0,110	0,073	-0,006	-0,031	-0,069	-0,083	-0,013	-0,093	0,002	-0,029	0,087	0,048	-0,058	-0,124	-0,079	0,034	0,047
**GDT**	0,105	0,203	0,008	0,108	0,126	0,123	0,079	-0,112	0,004	-0,016	0,025	0,114	0,006	0,043	0,071	0,027	0,086	-0,051	-0,081
	**LOW BETA**																		
**DDT_R**	0,121	-0,044	-0,076	-0,178	-0,139	-0,164	-0,079	-0,193	-0,092	-0,215	-0,041	-0,061	0,030	-0,076	-0,089	-0,131	-0,143	0,001	0,081
**DDT_L**	0,127	-0,086	0,05	-0,214	-0,166	-0,086	0,022	0,007	-0,102	-0,019	0,173	0,102	0,262	0,123	0,059	-0,057	0,001	0,153	0,213
**FPT**	0,098	-0,078	0,072	-0,094	-0,057	-0,018	0,001	0,034	0,037	0,009	0,130	0,121	0,233	0,171	0,099	-0,079	0,004	0,245	0,224
**DPT**	-0,022	-0,171	-0,083	-0,269	-0,204	-0,181	-0,022	-0,036	-0,111	-0,101	-0,017	-0,042	-0,104	0,020	-0,038	-0,217	-0,130	0,104	0,096
**aSpN**	0,047	0,178	0,204	0,069	-0,050	-0,042	0,017	-0,060	-0,020	-0,088	0,057	0,116	0,062	0,027	-0,045	-0,073	-0,079	0,046	0,053
**GDT**	-0,019	0,098	0,004	-0,117	-0,154	-0,051	-0,026	-0,149	-0,201	-0,076	-0,094	0,035	-0,077	-0,098	-0,099	-0,026	-0,094	-0,059	-0,041
	**MIDDLE BETA**																		
**DDT_R**	0,210	0,039	-0,086	0,006	0,017	-0,048	0,001	0,048	0,085	0,008	0,182	0,022	0,097	-0,023	0,061	0,041	0,004	0,122	0,129
**DDT_L**	0,142	-0,008	0,022	-0,017	0,012	0,040	0,031	0,143	0,040	0,170	0,189	0,057	0,207	0,097	0,153	0,074	0,090	0,160	0,162
**FPT**	-0,110	-0,084	0,070	0,014	0,054	0,039	0,162	0,093	0,117	0,038	0,096	0,204	0,190	0,289	0,078	0,109	0,090	0,148	-0,187
**DPT**	-0,010	-0,116	-0,052	-0,087	-0,098	-0,091	0,004	-0,001	-0,034	0,018	0,066	-0,072	0,110	0,014	0,090	-0,075	-0,033	0,152	0,030
**aSpN**	0,040	0,199	0,202	0,059	0,004	-0,058	-0,004	-0,021	0,032	-0,097	-0,031	0,113	0,146	0,059	0,042	-0,021	0,016	0,106	0,146
**GDT**	0,016	0,072	-0,047	-0,227	-0,181	-0,189	-0,107	-0,241	-0,212	-0,230	-0,068	0,122	-0,054	0,031	-0,166	-0,122	-0,176	-0,032	-0,024
	**HIGH BETA**																		
**DDT_R**	0,190	-0,010	-0,072	-0,033	-0,061	-0,081	-0,035	0,024	0,030	0,046	0,134	-0,014	0,038	-0,049	0,039	0,051	0,033	0,185	0,185
**DDT_L**	0,087	-0,130	-0,094	-0,140	-0,059	-0,057	-0,127	0,115	0,026	0,167	0,001	-0,064	0,138	0,062	0,087	0,004	0,093	0,195	0,218
**FPT**	0,021	-0,182	-0,760	-0,106	-0,101	-0,094	-0,147	0,037	0,021	0,101	0,004	-0,065	0,112	0,075	0,102	-0,017	0,067	***0,326***	0,203
**DPT**	-0,061	-0,263	-0,181	-0,176	-0,167	-0,181	-0,130	-0,019	-0,087	0,038	-0,069	-0,176	0,018	-0,03	0,018	-0,157	-0,058	0,196	0,760
**aSpN**	-0,013	-0,188	0,133	-0,002	-0,027	-0,066	-0,071	-0,137	-0,068	-0,206	-0,114	0,047	-0,004	-0,053	-0,091	-0,109	-0,141	-0,036	-0,039
**GDT**	0,032	0,003	-0,077	-0,190	-0,205	-0,204	-0,197	-0,268	***-0,324***	***-0,337***	-0,105	0,007	-0,158	-0,121	-0,272	-0,237	-0,292	-0,186	-0,095

DDT_R – Dichotic Digit Test for the right ear, DDT_L – Dichotic Digit Test for the left ear, FPT – Frequency Pattern Test, DPT – Duration Pattern Test, GDT – Gap Detection Test, aSpN – adaptive Speech in Noise Test
